# Supplementary material for: Diagnostic Approaches for Neuroendocrine Neoplasms of Unknown Primary (NEN-UPs) and Their Prognostic Relevance—A Retrospective, Long-Term Single-Center Experience
Source: Cancers (Basel). 2023 Aug 29;15(17):4316. doi: 10.3390/cancers15174316 (PMC10486951; doi:10.3390/cancers15174316)
Supplement: Supplementary file 1 [file cancers-15-04316-s001.zip › Supplementary Figure S1.pdf]

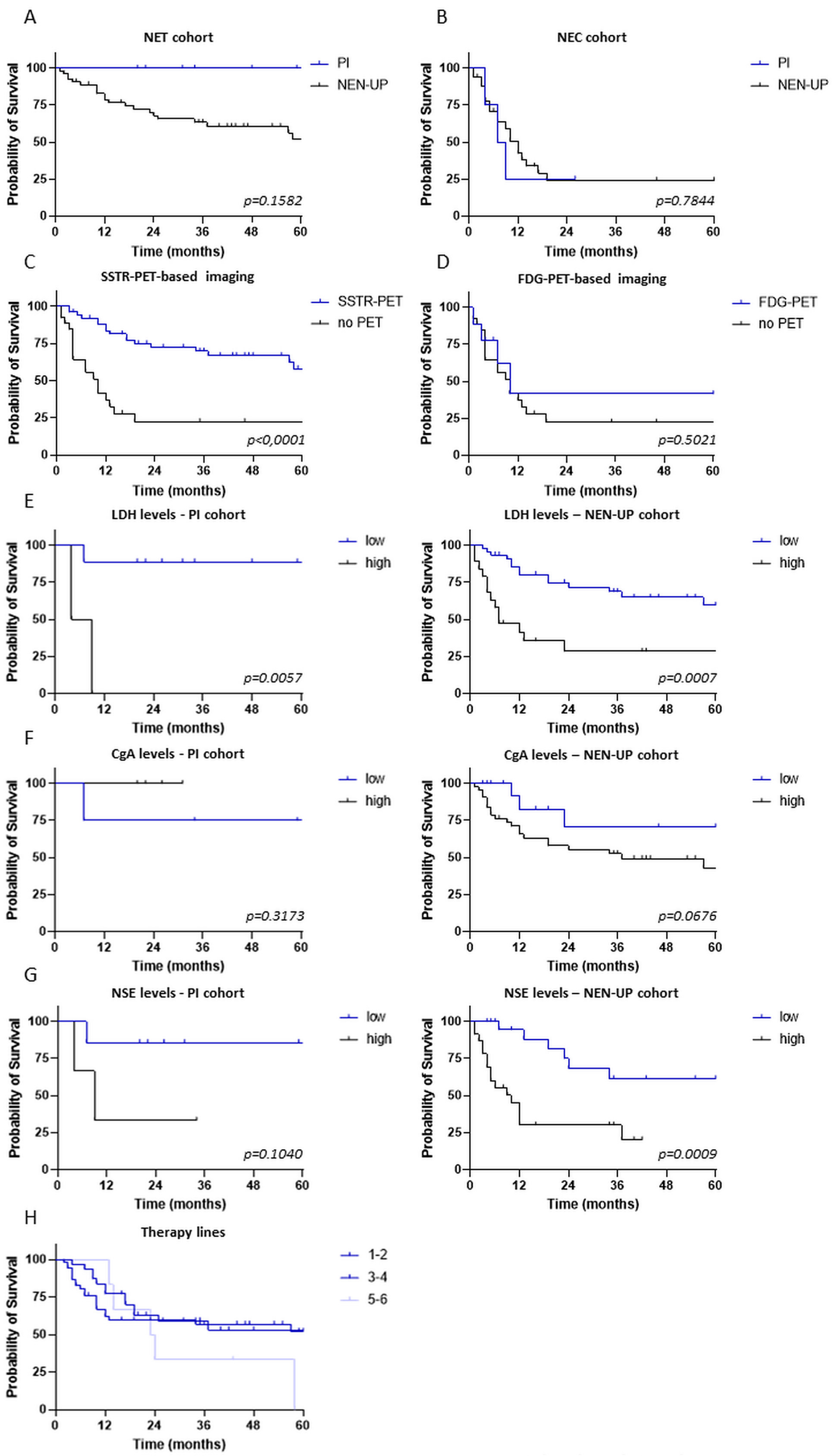

Figure S1. Prognostic role of clinical features, diagnostic instruments and therapeutic characteristics in different cohorts of patients with NEN-UP. Kaplan–Meier curves illustrate the probability of survival in the NET cohort (A), the NEC cohort (B) and depending on the performance of SSTR-PET-based imaging (C), FDG-PET-based imaging (D), serum levels of LDH in the PI and NEN-UP cohort (E), CgA in the PI and NEN-UP cohort (F), NSE in the PI and NEN-UP cohort (G) and therapy lines (H). Abbreviations: NET = neuroendocrine tumor; NEC = neuroendocrine carcinoma; NEN-UP = neuroendocrine neoplasm of unknown primary; PI = primary tumor site identified later on; SSTR = somatostatin receptor; FDG = fluorodeoxyglucose; CgA = chromogranin A; NSE = neuron-specific enolase; LDH = lactate dehydrogenase.
